# Changes in the mechanical properties and pressure pain thresholds of muscles in patients with fibromyalgia and their relationship with clinical parameters: a case-control study

**DOI:** 10.1007/s00421-026-06281-y

**Published:** 2026-06-06

**Authors:** Dilek Hande Esen, Serkan Taş, Mehmet Eroğlu

**Affiliations:** 1 Department of Therapy and Rehabilitation, Vocational School of Health Services, Toros University, 33140 Mersin, Turkey; 2 Department of Physical Therapy and Rehabilitation, Faculty of Health Sciences, Toros University, 33140 Mersin, Turkey; 3 Department of Physical Medicine and Rehabilitation, VM Medical Park Hospital, 33110 Mersin, Turkey

**Keywords:** Fibromyalgia syndrome, Stiffness, Tone, Elasticity, Pain sensitivity

## Abstract

**Purpose:**

The association between fibromyalgia and changes in muscle mechanical properties is unclear. The primary objective of this study was to compare the mechanical properties (tone, stiffness, and elasticity) of the upper trapezius (UT), extensor carpi radialis brevis (ECRB), and medial gastrocnemius (MG) muscles between patients with fibromyalgia (PwFM) and healthy controls. The secondary objective was to investigate the association between muscle mechanical properties and relevant clinical parameters.

**Methods:**

Twenty-nine women and two men with fibromyalgia, and 31 age-matched healthy controls were included in this study. Muscle mechanical properties were assessed using myotonometry (MyotonPRO^®^), pressure pain thresholds (PPTs) with a digital algometer (Commander Echo-Algometer^®^), pain intensity using a visual analog scale, and disease impact using the Revised Fibromyalgia Impact Questionnaire.

**Results:**

Muscle frequency (tone) and dynamic stiffness in the UT, ECRB, and MG were significantly higher in the PwFM group compared to controls (*p* < 0.05). Effect sizes were strong for the UT (**|**r**|**≥0.5), weak to moderate for the ECRB (**|**r**|**=0.293–0.306), and weak for the MG (|r|=0.293). No significant between-group differences were observed in logarithmic decrement (elasticity) (*p* > 0.05). In the PwFM group, a moderate positive correlation was found between the frequency of the MG muscle and its PPT (*r* = 0.508, *p* = 0.003).

**Conclusion:**

The present study revealed that the tone and stiffness of the UT, ECRB, and MG muscles were increased in PwFM. These findings indicate that altered muscle mechanical properties were associated with fibromyalgia and may warrant consideration in future research exploring clinical management approaches.

## Introduction

Fibromyalgia is a chronic disorder of multifactorial origin, marked by widespread musculoskeletal pain, heightened sensory sensitivity, persistent fatigue, sleep problems, and cognitive dysfunction (Filipovic et al. 2025; Siracusa et al. 2021). Its prevalence ranges from 2% to 3% worldwide, and it is more common in women (Meral et al. 2025). Individuals with this syndrome experience significant limitations in their daily activities and a decline in their overall quality of life. Although the etiology of fibromyalgia is not fully understood, abnormal pain processing in the central nervous system, peripheral nociceptive mechanisms, structural changes in muscle tissue, and psychosocial factors have been reported to play a role (Alonso-Blanco et al. 2011).

Recent studies have revealed that fibromyalgia is not limited to subjective complaints of pain and fatigue; it involves morphological and functional changes in the muscle tissue that can be objectively demonstrated (Gerdle et al. 2010; Meral et al. 2025; Mesci et al. 2023; Westgaard et al. 2013). For example, a study using ultrasound and blob analysis of the upper trapezius (UT) muscle demonstrated that patients with fibromyalgia (PwFM) had significantly higher muscle echo intensity and blob areas than healthy individuals, which were associated with degenerative changes in muscle tissue and possible fibrotic processes (Meral et al. 2025). Similarly, ultrasonographic studies have shown that PwFM has thinner gastrocnemius, UT, and upper extremity muscles than healthy individuals, and significant correlations have been found between muscle thickness and functional performance tests (handgrip strength and sit-to-stand test) (Mesci et al. 2023). In contrast, at the neuromuscular control level, a study using surface EMG found that PwFM showed less differential activation in the UT muscle, particularly exhibiting a more homogeneous but less flexible activation pattern under low load (Gerdle et al. 2010). Furthermore, it has been noted that UT muscle activity in PwFM increases significantly compared with in that healthy individuals under conditions that trigger mental stress and autonomic activation, reflecting stress-related physiological mechanisms (Westgaard et al. 2013).

The mechanical properties of muscles, including tone, stiffness, and elasticity, are fundamental determinants of joint stability and motor control (Stanev and Moustakas 2019). Furthermore, these properties constitute critical factors influencing movement performance and the capacity for force generation (Barron et al. 2022; Xu et al. 2021). In PwFM, the mechanical properties of muscles may be altered by mechanisms such as autonomic nervous system hyperactivity, inability of muscles to fully relax, disturbances in energy metabolism (e.g., high pyruvate, low ATP), structural changes, reduced blood flow, and increased central/peripheral sensitivity (Elvin et al. 2006; Gerdle et al. 2020; Marvulli et al. 2015; Westgaard et al. 2013). Based on this information, it can be considered that potential changes in muscle mechanical properties may affect muscle function and physical performance in PwFM. For example, reduced blood flow to muscles during exercise in PwFM may increase the tendency for spasms, leading to muscle stiffness and loss of conditioning (Gerdle et al. 2020). Indeed, muscle strength and aerobic capacity decrease in PwFM, while resistance to fatigue develops (Maquet et al. 2002; Marvulli et al. 2015). Therefore, examining muscle mechanical properties is important to understand the physiological reasons underlying functional performance loss (Mesci et al. 2023).

To the best of our knowledge, studies evaluating muscle mechanical properties in PwFM using objective methods are limited (Karayol and Karayol 2021; Navarro-Ledesma et al. 2023; Ornek et al. 2024). These studies reported findings such as decreased elasticity in the UT muscle (Navarro-Ledesma et al. 2023), increased stiffness in the rhomboideus major (Karayol and Karayol 2021), and no significant difference in the MG muscle (Ornek et al. 2024). However, the lack of a control group in these studies, the use of different assessment techniques, the variety of measurement sites, and conflicting results make it difficult to clarify the effect of fibromyalgia on muscle mechanical properties. The assessment of muscle mechanical properties in PwFM, alongside relevant clinical parameters, has the potential to substantially advance the understanding of the disease’s underlying pathophysiology. Furthermore, information about the level of stiffness in specific muscles enables the personalization of treatment and rehabilitation programs while also allowing for objective monitoring of the effectiveness of the interventions applied.

This study aimed to compare the mechanical properties of the UT, ECRB, and medial gastrocnemius (MG) muscles between PwFM and healthy controls. The secondary objective was to investigate the relationship between muscle mechanical properties and clinical parameters such as pressure pain thresholds (PPTs). We hypothesized that muscle stiffness and tone would increase, elasticity would decrease, and PPTs would decrease in PwFM, with muscle-specific variations most pronounced in the UT. We also predicted that these changes would show a positive correlation with clinical parameters such as pain intensity and disease impact level.

## Methods

### Study design

This case–control study was conducted between June and August 2025 at the Physical Therapy Outpatient Clinic of VM Medical Park Mersin Hospital. The study was performed in accordance with the Declaration of Helsinki and followed the STROBE reporting guidelines (von Elm et al. 2008). Ethical approval was obtained from the Non-Invasive Clinical Research Ethics Committee of X University (approval number 04–10, dated April 21, 2025). All participants provided written informed consent before participation.

### Sample size calculation

The sample size was calculated using G*Power 3.1.9.7 (Heinrich-Heine-Universität Düsseldorf, Germany), informed by muscle stiffness values obtained in a previous myotonometry-based investigation (Taş et al. 2024). A between-group difference of 30 N/m (equivalent to a 15% change) was defined as clinically meaningful, ensuring a statistical power of 95% with a type I error rate of 5%. Based on an assumed mean UT muscle stiffness of 202 N/m and a standard deviation of 29 N/m in the control group, the minimum sample size was calculated as 21 participants per group.

### Participants

**Fig. 1:**
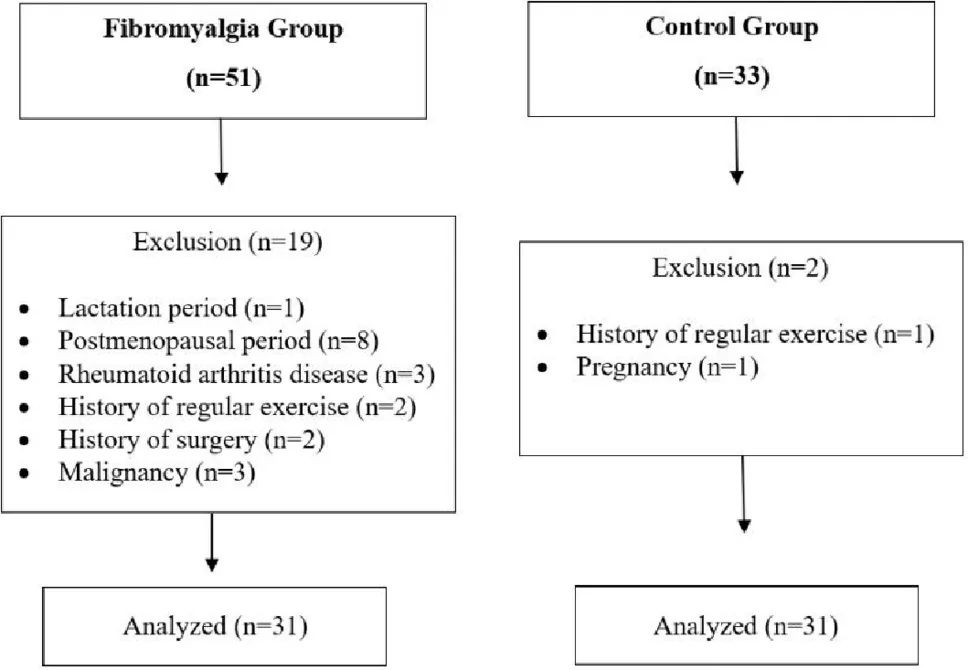
Flow diagram of participants

Thirty-one individuals with fibromyalgia (29 women, 2 men) meeting the 2016 ACR criteria (Wolfe et al. 2016) were enrolled, together with 31 age- and sex-matched healthy controls (median age: 38 years in both groups). All participants had a body mass index ≤ 30 kg/m² and had not engaged in any regular exercise or sports program within the past six months. The following exclusion criteria were applied to both groups: neurological, orthopedic, or other rheumatological conditions; history of major surgery or trauma; skin or systemic diseases; malignancy; pregnancy, postmenopausal status, or lactation; and cognitive or sensory impairments that could interfere with questionnaire completion. Specific to the patient group, individuals who had received fibromyalgia-specific treatment (pharmacological or physical therapy) within the past year were excluded. Furthermore, to avoid interference with PPT measurements, factors such as severe sleep disturbances and medications affecting the central nervous system (e.g., antidepressants and centrally acting analgesics) were considered exclusion criteria for all participants. Participants were recruited on a voluntary basis through open announcements posted on hospital bulletin boards and digital platforms, as well as through verbal information provided during routine outpatient visits, without any obligation to participate. To prevent any potential for coercion, it was clearly stated both verbally and in the written informed consent form that participation was strictly voluntary. Participants were informed that declining to participate would not affect their current or future medical care or their relationship with healthcare providers. After applying the predefined eligibility criteria, 31 eligible individuals from a pool of 51 previously diagnosed with fibromyalgia were included in the patient group, while the control group consisted of 31 healthy individuals (Fig. 1).

### Assessment procedure

The study groups were defined as independent variables. Dependent variables included Revised Fibromyalgia Impact Questionnaire (RFIQ) scores, visual analogue scale scores, mechanical properties (tone, stiffness, elasticity), and PPTs of the UT, ECRB, and MG muscles. The selection of the UT, ECRB, and MG muscles was based on their representative roles across different anatomical regions and functional demands. The UT was selected due to its high clinical relevance as a commonly affected muscle and frequently assessed tender point in patients with fibromyalgia, as well as its reported structural and functional alterations (Gerdle et al. 2010; Westgaard et al. 2013; Meral et al. 2025). The ECRB was chosen to represent distal upper limb involvement, which plays a key role in functional activities such as handgrip and has been previously evaluated using myotonometric methods (Çevik Saldıran et al. 2022). Finally, the MG was included as a representative of weight-bearing lower limb muscles, allowing assessment of regional differences and supported by studies reporting structural and functional changes in this muscle group in fibromyalgia (Mesci et al. 2023; Ornek et al. 2024). All measurements were performed on the dominant limb by same researcher in a single session, following a standardised protocol to ensure consistency in positioning and instructions. Upper limb dominance was defined as the hand used for writing (Park 2013), while lower limb dominance was determined by the preferred leg used for kicking a ball (Esen et al. 2025). Prior to the assessment, participants underwent a brief 5-minute rest to minimize physiological variability. Measurements were conducted while participants remained in a the fully resting position. Sociodemographic data and clinical questionnaires (VAS and RFIQ) were collected prior to the physical assessments by a member of the research team who was not involved in the measurement procedures. The researcher performing the muscle mechanical property and PPT measurements was blinded to group allocation and had no access to questionnaire data or participant diagnosis. Muscle mechanical properties were assessed using a myotonometer (MyotonPRO^®^, Myoton Ltd., Tallinn, Estonia), and PPTs were measured with a digital algometer (Commander Echo-Algometer^®^, JTECH Medical, Midvale, USA). Participants were instructed to avoid caffeine, alcohol, medication and intense physical activity for 48 h prior to testing.

#### Visual analogue scale

The evaluation of pain intensity was performed with a 10‑cm visual analogue scale (VAS), ranging from 0 (absence of pain) to 10 (maximum pain). Participants reported their average pain during the past week, and values were measured in centimetres. The VAS is recognized as a reliable instrument for evaluating pain in chronic disorders such as fibromyalgia (Boonstra et al. 2008).

#### Revised fibromyalgia impact questionnaire

To assess symptom severity in PwFM, the Turkish version of the RFIQ, which has previously been validated and demonstrated reliability, was used. This tool consists of 21 items divided into three domains: physical functioning, overall impact, and symptoms. Each domain is scored using a 0–10 numeric scale, resulting in an overall score ranging from 0 to 100. Higher total scores reflect greater disability associated with fibromyalgia (Ediz et al. 2011).

#### Measurement of the mechanical properties of muscles

Muscle mechanical properties were measured using a validated myotonometer (MyotonPRO^®^, Myoton Ltd., Tallinn, Estonia) in accordance with established protocols (Çevik Saldıran et al. 2022; Liu et al. 2018; Taş et al. 2022). Prior to measurement, test points on the muscle belly were marked, and a brief (15 ms) mechanical impulse was applied perpendicularly using a pressure of up to 0.6 N. Measurements were repeated three times with 30-second rest intervals, and the mean value was recorded.

**Fig. 2:**
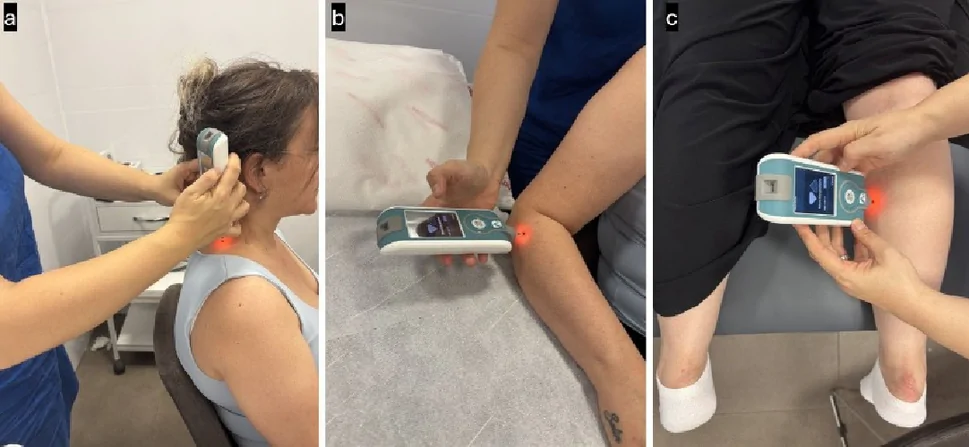
Measurement of the mechanical properties of the upper trapezius (**a**), extensor carpi radialis brevis (**b**) and medial gastrocnemius (**c**) muscle

Assessment of the UT muscle was performed with participants seated, and the probe was applied at the anatomical midpoint between the C7 spinous process and the lateral acromion (Liu et al. 2018). Participants were seated with back support during ECRB assessment, maintaining slight shoulder abduction (10–20°), 90° elbow flexion, forearm pronation, and neutral wrist position. The measurement site was located on the dorsal surface approximately 4 cm distal to the lateral epicondyle, with wrist extension and radial deviation performed to confirm anatomical reference (Çevik Saldıran et al. 2022). The MG muscle was assessed with participants in a prone position, with their feet hanging freely off the edge of the bed. The measurement site was determined at 30% of the lower leg length, calculated along the line from the popliteal crease to the lateral malleolus (Taş et al. 2022) (Fig. 2).

#### Measurement of the pressure-pain threshold

The PPTs of the target muscles were measured using a validated digital algometer (Commander Echo-Algometer^®^, JTECH Medical, Midvale, USA) (Park et al. 2011). Participant positioning and measurement sites followed the myotonometry protocol. After placing the algometer tip perpendicularly at the designated point, the pressure was gradually increased and participants were asked to say ‘stop’ as soon as they felt pain. Immediately after this signal, the device was released and the force value displayed on the screen was recorded. All measurements were performed using a 1 cm² probe head, with a standardized pressure increase rate of 1 kg/cm² per second. Participants were not permitted to view the algometer screen at any stage of the procedure. Prior to data collection, participants received detailed instructions and completed a familiarization trial. To minimize the influence on pain sensitivity, a 30-second rest interval was provided between consecutive measurements. The mean of three repeated measurements at each site was recorded in kg/cm². Lower PPTs were interpreted as indicating higher pain sensitivity (Navarro-Ledesma et al. 2023).

### Statistical analysis

Statistical analyses were performed using SPSS Windows version 27.0 (IBM Corp., Armonk, NY, USA) by a researcher blinded to the data collection. The non-normal distribution of the variables was confirmed using the Kolmogorov–Smirnov test and visual methods (histograms and probability plots). Numerical data were reported as median and interquartile range (25th–75th percentile), and categorical data as frequency (n) and percentage (%). Group comparisons were conducted using the chi-square test for categorical variables and the Mann–Whitney U test for numerical variables. The primary outcome of the study was the difference in UT, ECRB, and MG muscle mechanical properties (tone, stiffness, and elasticity) between PwFM and healthy controls. Secondary outcomes included differences in PPTs of the UT, ECRB, and MG muscles, as well as the relationships between these properties and clinical parameters such as pain intensity and disease impact level. In addition, analysis of covariance (ANCOVA) was performed to compare MG outcomes between groups, adjusting for lower extremity dominance as a covariate. For the Mann–Whitney U test, effect sizes (r) were classified as small (**|**r**|**>0.1), moderate (**|**r**|**≥0.3), and large (**|**r**|**≥0.5) (Esen et al. 2025). Correlations were examined using Spearman’s test and categorized as weak (*r* < 0.3), moderate (*r* = 0.3–0.5), strong (*r* = 0.5–0.7), or very strong (*r* > 0.7) (Mukaka 2012). Statistical significance was set at *p* < 0.05.

## Results

The sample comprised 29 women and 2 men with fibromyalgia and 31 healthy controls matched for age and gender.

### Participant characteristics

There was no significant difference between the groups in terms of age (*p* = 0.910), height (*p* = 0.372), weight (*p* = 0.568), body mass index (*p* = 0.508), upper extremity dominance (*p* = 0.612), marital status (*p* = 0.317), education level (*p* = 0.993), occupational groups (*p* = 0.788), and work type (*p* = 0.430). However, a significant difference was observed between the groups in terms of lower extremity dominance (*p* = 0.022); left dominance was more common in the fibromyalgia group (32.3% in PwFM vs. 6.5% in control), while right dominance was significantly higher in the control group. The most common level of education in both groups was university graduation (64.5% in PwFM and 64.5% in controls). The average duration of pain in the fibromyalgia group was reported as 24 months (4–48 months). The mean VAS score was 8.50 cm (7.10–9.60 cm), and the RFIQ score was 70.30 (61.80–78.60). The sociodemographic and clinical profiles of the groups are detailed in Table 1.

**Table 1:** Comparison of demographic and clinical characteristics between fibromyalgia patients and healthy controls

Descriptive parameters	Fibromyalgia group (*n* = 31)	Control group (*n* = 31)	*P* value
Demographics characteristics			
Age, years	38 (32–44)	38 (32–42)	0.910^a^
Height, m	1.65 (1.60–1.67)	1.63 (1.58–1.68)	0.372^a^
Weight, kg	60 (56–68)	61 (55–70)	0.568^a^
Body mass index, kg/m^2^	22.32 (20.96–24.98)	22.64 (21.48–25.95)	0.508^a^
Gender			
Female, n (%)	29 (93.5%)	29 (93.5%)	0.694^b^
Male, n (%)	2 (6.5%)	2 (93.5%)	
Dominant upper limb, n (%)			
Right	28 (90.3%)	30 (96.8%)	0.612^b^
Left	3 (9.7%)	1 (3.2%)	
Dominant lower limb, n (%)			
Right	21 (67.7%)	29 (93.5%)	**0.022** ^*b^
Left	10 (32.3%)	2 (6.5%)	
Marital status, n (%)			
Single	6 (19.47%)	7 (22.6%)	0.317^b^
Married	19 (61.3%)	22 (71.0%)	
Divorced	6 (19.4%)	2 (6.5%)	
Education level, n (%)			
Primary school	1 (3.2%)	1 (3.2%)	0.993^b^
Middle school	2 (6.5%)	2 (6.5%)	
High school	4 (12.9%)	5 (16.1%)	
University	20 (64.5%)	20 (64.5%)	
Postgraduate	4 (12.9%)	3 (6.7%)	
Occupation, n (%)			
Civil servant	8 (25.8%)	6 (19.4%)	0.788^b^
Worker	1 (3.2%)	1 (3.2%)	
Private sector employee	18 (58.1%)	17 (54.8%)	
Retired	0 (0.0%)	1 (3.2%)	
Housewife	4 (12.9%)	6 (19.4%)	
Work type, n (%)			
Desk job	12 (38.7%)	9 (29.0%)	0.430^b^
Standing work	14 (45.2%)	19 (61.3%)	
Manual laborer	5 (16.1%)	3 (9.7%)	
Clinical characteristic			
Pain duration, months	24 (4–48)	NA	
VAS, cm	8.50 (7.10–9.60)	NA	
RFIQ	70.30 (61.80–78.60)	NA	

Quantitative variables are presented as median (interquartile range 25–75%)

*NA* not applicable, *VAS* visual analogue scale, *RFIQ* revised fibromyalgia impact questionnaire

*p < 0.05

^a^Mann–Whitney U test

^b^Chi-Square test. n, number of participants

### Group comparison of muscle mechanical properties and pressure-pain threshold

In the PwFM, the frequency values of the UT (*p* < 0.001), ECRB (*p* = 0.020), and MG (*p* = 0.017) muscles were significantly higher compared to the control group. Similarly, the dynamic stiffness values of UT (*p* < 0.001), ECRB (*p* = 0.042), and MG (*p* = 0.009) muscles were also significantly higher in the PwFM. The effect size (r) values for these differences indicate a strong effect for UT (|r|=0.561–0.571); a weak-to-moderate effect for ECRB (tone: |r|=0.306; stiffness: |r|=0.281); and a weak effect for MG (|r|=0.293). No significant differences were found between groups in terms of the logarithmic decrement parameters (*p* > 0.05). When lower extremity dominance was included as a covariate in the analysis, the group differences in MG frequency (F = 10.525, *p* = 0.002, partial η² = 0.161) and dynamic stiffness (F = 9.699, *p* = 0.003, partial η² = 0.150) remained statistically significant. Lower extremity dominance itself was not a significant predictor (for frequency: *p* = 0.338; for dynamic stiffness: *p* = 0.516).

In PPT measurements, significantly lower PPTs were observed in the fibromyalgia group across all muscles (*p* < 0.001), supported by large effect sizes (|r|=0.782–0.827). After adjusting for lower extremity dominance, a significant group difference was observed in MG pressure pain thresholds (F = 46.122, *p* < 0.001, partial η² = 0.456). Lower extremity dominance was not a significant covariate (*p* = 0.659).Group comparisons of muscle mechanical properties and PPT values are summarized in Table 2.

**Table 2:** Comparison of muscle mechanical properties and pressure pain threshold of selected muscles between fibromyalgia and control groups

Variables	Fibromyalgia group (*n* = 31)	Control group (*n* = 31)	*P*-value	Effect size (*r*)
Frequency (Hz)				
Upper trapezius	17.57 (16.70–18.97)	16.10 (14.97–16.90)	< 0.001*	– 0.571
Extensor carpi radialis brevis	15.43 (14.67–17.97)	14.90 (13.13–16.30)	0.020*	– 0.306
Medial gastrocnemius	15.03 (13.53–15.57)	14.20 (13.43–14.90)	0.017*	– 0.293
Dynamic stiffness (N/m)				
Upper trapezius	321 (306–372)	288 (264–299)	< 0.001*	– 0.561
Extensor carpi radialis brevis	287 (262–317)	267 (238–291)	0.042*	– 0.281
Medial gastrocnemius	256 (238–279)	244 (225–259)	0.009*	– 0.293
Logarithmic decrement				
Upper trapezius	1.16 (1.05–1.30)	1.18 (1.06–1.31)	0.637	– 0.008
Extensor carpi radialis brevis	1.63 (1.49–1.85)	1.60 (1.52–1.83)	0.921	0.000
Medial gastrocnemius	1.16 (1.06–1.29)	1.13 (1.01–1.28)	0.822	– 0.044
Pressure pain threshold				
Upper trapezius	1.50 (1.30–1.80)	2.70 (2.20–2.80)	< 0.001*	– 0.782
Extensor carpi radialis brevis	1.60 (1.20–1.90)	2.60 (2.20-3.00)	< 0.001*	– 0.789
Medial gastrocnemius	1.50 (1.30–1.80)	2.70 (2.30–3.70)	< 0.001*	– 0.827

*p < 0.05, Mann-Whitney U test. Quantitative variables are presented as median (interquartile range 25–75%)

### Correlation analysis results

In PwFM, the MG muscle showed a moderate positive correlation between frequency and PPT values (*r* = 0.508, *p* = 0.003). whereas no significant associations were observed for the other muscles (*p* > 0.05). The findings of the correlation analysis are shown in Table 3.

**Table 3:** Correlations between clinical variables and muscle mechanical properties in selected muscles in patients with fibromyalgia

Variables		Frequency (Hz)			Dynamic stiffness (N/m)			Logarithmic decrement		
		UT	ECRB	MG	UT	ECRB	MG	UT	ECRB	MG
Age, years	r	0.135	0.156	0.142	0.154	0.163	0.190	0.055	0.238	0.180
	*p*	0.461	0.393	0.437	0.401	0.372	0.297	0.764	0.189	0.325
Height, cm	r	0.167	– 0.031	0.087	0.178	– 0.035	0.087	– 0.147	– 0.196	– 0.098
	*p*	0.362	0.871	0.652	0.329	0.858	0.652	0.422	0.309	0.614
Weight, kg	r	0.139	– 0.065	0.202	0.178	0.114	0.202	0.064	0.109	0.061
	*p*	0.448	0.737	0.293	0.329	0.554	0.293	0.729	0.575	0.753
BMI, kg/m^2^	r	0.005	– 0.058	0.153	0.041	0.146	0.153	0.074	0.177	0.102
	*p*	0.979	0.765	0.429	0.825	0.450	0.429	0.688	0.358	0.600
Pain duration, months	r	– 0.002	0.244	– 0.102	0.032	0.230	0.004	– 0.267	– 0.245	0.108
	*p*	0.990	0.187	0.585	0.866	0.213	0.982	0.147	0.183	0.565
VAS, cm	r	– 0.039	0.087	0.125	0.061	0.231	0.091	– 0.051	0.059	– 0.036
	*p*	0.836	0.641	0.502	0.744	0.212	0.628	0.786	0.751	0.847
FIQ	r	– 0.055	0.165	0.096	0.069	0.234	– 0.131	– 0.092	– 0.129	0.078
	*p*	0.770	0.375	0.606	0.714	0.205	0.482	0.623	0.490	0.676
PPT-UT	r	– 0.010	– 0.061	0.314	0.020	0.067	0.294	0.043	0.017	0.061
	*p*	0.956	0.740	0.080	0.912	0.717	0.103	0.817	0.925	0.741
PPT-ECRB	r	0.118	0.072	0.313	0.127	0.204	0.231	0.071	-0.085	0.403
	*p*	0.518	0.695	0.081	0.488	0.262	0.202	0.701	0.643	0.300
PPT-MG	r	– 0.158	– 0.238	0.508*	– 0.153	– 0.225	0.321	– 0.143	– 0.159	– 0.056
	*p*	0.387	0.190	0.003	0.404	0.216	0.073	0.436	0.385	0.763

*p < 0.05. r, Spearman’s correlation coefficient

*UT* upper trapezius, *ECRB* extensor carpi radialis brevis, *MG* medial gastrocnemius, *BMI* body mass index, *VAS* visual analogue scale, *FIQ* fibromyalgia impact questionnaire, *PPT* pressure pain threshold

## Discussion

In the present study, the tone, stiffness, and elasticity of the UT, ECRB, and MG muscles were compared between PwFM and healthy controls. The findings revealed that tone and stiffness levels were significantly increased in all examined muscles in PwFM, while PPT values were markedly reduced; however, no significant difference was observed in elasticity. These results generally support the study’s primery hypothesis, and the effect size analysis indicates that mechanical alterations were pronounced in the UT muscle compared to the others. However, contrary to our hypothesis, correlation analyses showed no significant relationship between other muscle mechanical properties and PTTs, pain intensity, or disease impact level in the PwFM group, except for the moderate positive correlation observed between MG muscle frequency and PPT.

In this study, the association of fibromyalgia with increased tone and stiffness in the UT, ECRB, and MG muscles can be explained by dysfunctions in neuromuscular control mechanisms, excessive sympathetic nervous system activation, and impaired muscle energy metabolism (Cordero et al. 2010; Gerdle et al. 2010, 2020; Karayol and Karayol 2021; Navarro-Ledesma et al. 2023; Westgaard et al. 2013). Previous studies have reported that the normal relaxation capacity of muscles is impaired in PwFM; muscles may remain continuously in a low-level active or contracted state, which may increase muscle tone and the sensation of stiffness (Elert et al. 2001; Westgaard et al. 2013; Zetterman et al. 2021). Westgaard et al. (2013) found that UT muscle activity and sympathetic activation levels were higher at rest in PwFM compared to healthy controls. They explained this by suggesting that PwFM may exhibit increased muscle activity in response to stressful stimuli due to autonomic nervous system dysregulation. Similarly, Zetterman et al. (2021) reported that muscles in PwFM remain active for longer periods without transitioning into a resting state. On the other hand, some researchers have linked the excessive autonomic nervous system activation observed in PwFM to alterations in neurotransmitter levels such as norepinephrine and serotonin. This may lead to excessive muscle contraction, thereby increasing tonic muscle activity and passive muscle stiffness (Marvulli et al. 2015). Moreover, impaired muscle energy metabolism and ATP deficiency due to mitochondrial dysfunction in PwFM may also contribute to increased muscle tone and stiffness by making muscle relaxation more difficult. Cordero et al. (2010) demonstrated a reduction in the energy production capacity of mitochondria in PwFM. Similarly, some studies have reported that the energy production of mitochondria in the UT muscle of PwFM is insufficient compared to healthy controls, and that interstitial lactate and pyruvate concentrations are significantly higher (Gerdle et al. 2010, 2020).

A notable finding of the present study was that muscle elasticity in PwFM was similar to that of healthy individuals. This suggests that increased tone and stiffness may be more closely related to active neuromuscular regulation dysfunctions rather than a loss of elasticity (Feng et al. 2018; Stanev et al., 2019). While passive elasticity largely depends on matrix components such as collagen, elastin, and titin, both muscle tone and stiffness are influenced by passive tissues as well as active processes such as sympathetic activation and motor unit firing rates (Akhtar et al. 2011; Feng et al. 2018; Maciejewska-Skrendo et al. 2020). Supporting this perspective, Navarro-Ledesma et al. (2022) reported that the loss of elasticity related to increased stiffness in PwFM may be associated with sympathetic nervous system dysfunction. Karayol and Karayol (2021) also suggested that increased stiffness in the rhomboideus major muscle may result from spasticity in PwFM. Conversely, Ornek et al. (2024) found no significant differences between PwFM and healthy controls in terms of tone, stiffness, and elasticity of the MG muscle. These conflicting findings may result from different measurement techniques, and variability in the tissues examined. Future investigations are needed to elucidate the underlying mechanisms linking fibromyalgia with biomarkers of muscle elasticity.

Similar to previous studies, the findings of this study indicate that PPT values were significantly lower in all muscles examined in PwFM, demonstrating increased sensitivity of these muscles to mechanical stimulation. The support of this finding by high effect sizes was clinically consistent and points to a widespread increase in pain in the muscles of PwFM (Alonso-Blanco et al. 2011; Gerdle et al. 2010; Navarro-Ledesma et al. 2023; Lei et al. 2025; Úbeda-D’Ocasar et al. 2021). However, contrary to the study by Navarro-Ledesma et al. (2023), which reported a correlation between reduced PPTs and increased stiffness in the UT muscle, the present study found no significant relationship between UT mechanical properties and PPT values.

The finding that only the MG demonstrated a significant correlation between muscle frequency and PPT should be interpreted with caution. While this may partly reflect muscle-specific adaptations related to its weight-bearing function, current evidence indicates that the relationship between muscle mechanical properties and pain sensitivity is not consistent across regions or conditions. For instance, Proulx et al. (2023) reported increased muscle stiffness and reduced PPT in women with chronic pelvic pain, yet no significant association between these variables was found. Similarly, Seidel et al. (2023) observed increased stiffness in the upper trapezius without corresponding changes in PPT. In fibromyalgia, Navarro-Ledesma et al. (2023) identified a significant association only in the dominant upper trapezius, with no correlations across other tender points. These findings collectively suggest that muscle mechanical properties and pain sensitivity may represent partially independent or region-specific phenomena. In line with this, the absence of significant correlations between most clinical variables (e.g., age, BMI, pain intensity, and disease impact) and muscle mechanical properties in the present study further supports the notion that these relationships are not straightforward but likely influenced by multifactorial mechanisms, including central sensitization. Therefore, the isolated correlation observed in the MG may reflect a localized or condition-specific adaptation, but it may also represent a statistical finding rather than a consistent physiological pattern. Furthermore, although statistically significant between-group differences were observed, their clinical relevance remains uncertain due to the lack of well-established minimal clinically important differences (MCID) for myotonometry-derived parameters. Future research is required to clarify these relationships and establish clinically meaningful thresholds.

### Strengths and limitations

One of the strengths of the present study was the sociodemographic similarity between the fibromyalgia and control groups. This similarity supports the interpretation that differences in muscle tone, stiffness, and mechanical sensitivity are specific to the disease. Furthermore, this study was the first to demonstrate the effect of fibromyalgia on the mechanical properties and PPTs of the ECRB and MG muscles. However, the fact that the proportion of adult female participants was much higher than that of male participants limited the generalizability of the findings to men and other age groups. Additionally, the fact that all measurements were taken in the resting position and only selected muscle groups were evaluated represents methodological limitations.

## Conclusion

The current study revealed increased tone and stiffness in the UT, ECRB, and MG muscles of PwFM, along with a significant reduction in PPT values. These findings suggest that fibromyalgia is not limited to subjective symptoms but may involve alterations in the passive mechanical properties of muscle tissue and pain sensitivity. The results highlight the importance of considering muscle mechanics and mechanical sensitivity in the diagnosis and management of fibromyalgia, and suggest that interventions targeting muscle relaxation, alongside pain control, may offer clinically meaningful benefits.

## Data Availability

The datasets generated and/or analyzed during the current study are not publicly available due to ethical restrictions and to ensure the confidentiality of participants. However, they can be obtained from the corresponding author upon reasonable request.

## Abbreviations

ANCOVA: Analysis of covariance
ECRB: Extensor carpi radialis brevis
MG: Medial gastrocnemius
UT: Upper trapezius
PPT: Pressure pain threshold
PwFM: Patients with fibromyalgia
RFIQ: Revised fibromyalgia impact questionnaire
VAS: Visual analogue scale
